# The Hypervariable *Tpr* Multigene Family of *Theileria* Parasites, Defined by a Conserved, Membrane-Associated, C-Terminal Domain, Includes Several Copies with Defined Orthology Between Species

**DOI:** 10.1007/s00239-023-10142-z

**Published:** 2023-11-28

**Authors:** Nicholas C. Palmateer, James B. Munro, Sushma Nagaraj, Jonathan Crabtree, Roger Pelle, Luke Tallon, Vish Nene, Richard Bishop, Joana C. Silva

**Affiliations:** 1 Institute for Genome Sciences, University of Maryland School of Medicine, Baltimore, MD USA; 2 International Livestock Research Institute, Nairobi, Kenya; 3 Department of Veterinary Microbiology and Pathology, Washington State University, Pullman, WA USA; 4 Department of Microbiology and Immunology, University of Maryland School of Medicine, Baltimore, MD USA; 5 Global Health and Tropical Medicine, GHTM, Instituto de Higiene E Medicina Tropical, IHMT, Universidade NOVA de Lisboa, UNL, Lisbon, Portugal

**Keywords:** Multigene family, *Theileria parva*, Lineage-specific expansion, Assembly gaps

## Abstract

Multigene families often play an important role in host-parasite interactions. One of the largest multigene families in *Theileria parva*, the causative agent of East Coast fever, is the *T. parva* repeat (*Tpr*) gene family. The function of the putative Tpr proteins remains unknown. The initial publication of the *T. parva* reference genome identified 39 *Tpr* family open reading frames (ORFs) sharing a conserved C-terminal domain. Twenty-eight of these are clustered in a central region of chromosome 3, termed the “*Tpr* locus”, while others are dispersed throughout all four nuclear chromosomes. The *Tpr* locus contains three of the four assembly gaps remaining in the genome, suggesting the presence of additional, as yet uncharacterized, *Tpr* gene copies. Here, we describe the use of long-read sequencing to attempt to close the gaps in the reference assembly of *T. parva* (located among multigene families clusters), characterize the full complement of *Tpr* family ORFs in the *T. parva* reference genome, and evaluate their evolutionary relationship with *Tpr* homologs in other *Theileria* species. We identify three new *Tpr* family genes in the *T. parva* reference genome and show that sequence similarity among paralogs in the *Tpr* locus is significantly higher than between genes outside the *Tpr* locus. We also identify sequences homologous to the conserved C-terminal domain in five additional *Theileria* species. Using these sequences, we show that the evolution of this gene family involves conservation of a few orthologs across species, combined with gene gains/losses, and species-specific expansions.

**Supplementary Information:**

The online version contains supplementary material available at 10.1007/s00239-023-10142-z.

## Introduction

Rapidly evolving multigene families, comprised of sets of genes descended from a common ancestral gene and which therefore share significant sequence similarity (Nei and Rooney 2005), are a typical genomic feature of many species of parasitic protozoa. Among these are species in the phylum Apicomplexa, which encompass species that cause disease and death in humans and other mammals, including livestock (Schnittger et al. 2022; Sharp et al. 2020; Sibley 2003). These multigene families frequently encode proteins that enable parasites to infect host cells, avoid innate and acquired immune responses, and minimize damage until the parasites can be transmitted to a new host (Reid 2015). Multigene families have been observed in most Apicomplexa for which genome sequences have been determined. Multigene families can be present across multiple taxa or be unique to a single species (Kissinger and DeBarry 2011). Lineage-specific multigene families are important to parasitic adaptation to and survival in mammalian hosts, and evolve rapidly, as suggested by a higher level of sequence divergence in genes with higher degree of lineage specificity among sister species (Kuo and Kissinger 2008).

One of the most studied and best characterized multigene families among Apicomplexa is the *var* gene family, from the parasite *Plasmodium falciparum*, which causes malaria in humans. The *var* genes encode variant antigens displayed on the surface of infected red blood cells (Leech et al. 1984), which are involved in malaria pathogenesis and immune system evasion (Gomes et al. 2016). Other examples of multigene families in apicomplexan organisms include the *smORF* gene family in *Babesia bovis* (Brayton et al. 2007), *ropk* in *Toxoplasma gondii* and other closely related coccidian parasites (Talevich and Kannan 2013), and the *ema* gene family present in *Theileria equi* (Kappmeyer et al. 2012) and *Theileria haynei* (Knowles et al. 2018). These gene families demonstrate a wide range of functions in different species within the phylum Apicomplexa, including facilitating the sequestration of infected cells to avoid splenic clearance (Seydel et al. 2006), virulence factors that enhance parasite survival against host interferon gamma-stimulated innate immune effector cells (Fox et al. 2016), and disruption of lytic enzymatic functions of the host cell in *T. equi* (Wise et al. 2019).

*Theileria parva* is an apicomplexan parasite whose genome contains several multigene families. *T. parva* causes East Coast fever, a fatal disease of cattle in Sub-Saharan Africa, which has been estimated to result in the death of ~ 1 million animals per year (Norval et al. 1991). The best characterized multigene families in *T. parva* are the sub-telomeric variable secreted protein (*SVSP*) gene family (Gardner et al. 2005), the *TpHN* gene family, which contains an orthologous gene family *TashAT* in *Theileria annulata* (Swan et al. 1999), and the *Tpr* gene family (Bishop et al. 1997; Weir et al. 2010), which also has homologs in *T. annulata*, designated as the *Tar* gene family (Pain et al. 2005).

*Tpr* gene family is defined by the presence of a conserved sequence in the 3′ end of each gene, leading to a conserved C-terminal domain in this single exon gene family (Gardner et al. 2005). The initial reference genome contained 39 hypothetical protein-coding genes that were annotated as belonging to the *Tpr* gene family. Of these 39 genes, 28 are tandemly arrayed on chromosome 3, in a region known as the *Tpr* locus, located ~ 570 Kb from one telomere and ~ 1.3 Mb from the other. While the genes described from this locus are based on a tandem array of open reading frames (ORFs), many copies are partial, in that they lack in-frame methionine codons at the 5′ end (Baylis et al. 1991; Gardner et al. 2005). The 5′ end of the genes lack detectable similarity between *Tpr* paralogs within a single genome, or between selected *T. parva* isolates based on Southern blot data (Bishop et al. 1997); in contrast, the 3′ end of the genes are highly conserved, encoding several predicted transmembrane domains. The other eleven loci annotated as *Tpr* genes, based on the presence of the encoded C-terminal domain, are distributed throughout the four nuclear chromosomes. These ORFs outside of the *Tpr* locus also maintain the conserved 3′ end, while demonstrating variability, between *T. parva* isolates, in length and sequence at the 5′ end.

Tpr proteins are predicted to be integral membrane proteins, based on the presence of multiple transmembrane domains (Bishop et al. 1997; Weir et al. 2010), but their function remains unknown. However, by analogy with other rapidly evolving multicopy gene families in pathogens, *Tpr* genes may be important for rapid generation of genetic diversity and host adaptation (Wilson et al. 2005) and/or in the evasion of host’s immune system (Gomes et al. 2016). Analysis of lineage-specific genes among a subset of Apicomplexa species indicated that the *Tpr* gene is specific to the genus *Theileria* and, further, that some genes are species-specific to either *T. parva* or *T. annulata* (Kuo and Kissinger 2008). Pain et al. (2005) showed that despite *Tpr* gene homologs occurring in the genomes of both of these *Theileria* species, the tandemly arrayed *Tpr* locus was present only in *T. parva* (Muguga), since a corresponding gene expansion in the orthologous region of chromosome 3 was absent from the *T. annulata* reference genome.

The *T. parva* genome assembly was published in 2005 and consisted of four nuclear chromosomes, plus the apicoplast and mitochondrial genomes (Gardner et al. 2005). Chromosomes 1 and 2 were each assembled in a single contig, while chromosomes 3 and 4 contained three and one gaps, respectively. The four gaps in the *T. parva* genome assembly are all located in regions that contain several members of a multigene family. These regions were particularly difficult to assemble after the initial sequencing of the genome due to the large number of consecutive genes present within them and to the high degree of sequence similarity among those gene copies. The gap in chromosome 4 is within a region of SVSP-encoding genes, and the three gaps in chromosome 3 fall within the *Tpr* locus.

Here, using a PacBio long-read sequencing approach, we sought to identify additional members of multigene families in the *T. parva* genome assembly by closing the remaining gaps in the current reference assembly. We closed one of three gaps in the *Tpr* locus and identified an additional copy of the *Tpr* multigene family. We also closed the gap in chromosome 4, resulting in three of the four nuclear chromosomes in the assembly now being complete. Additionally, we conducted a detailed analysis of the relationships among *Tpr*-encoded proteins. We completed a comprehensive examination of *Tpr* organization within the reference strain (Muguga), and demonstrated the existence of genes with a high degree of sequence similarity to those in the *Tpr* locus in *T. parva* strains other than the reference Muguga strain. We further analyzed the conserved C-terminal core domain of the resulting proteins across multiple species of *Theileria.* We sought to understand the evolutionary forces that led to the large *Tpr* gene expansion in chromosome 3 that appears to be specific to *T. parva*. Our analysis reveals distinct levels of *Tpr* gene family sequence similarity, both within the *T. parva* genome and across multiple *Theileria* species. This difference in similarity is a hallmark of varying levels of evolutionary conservation and suggests distinct functions within this multigene family, despite the ubiquitous presence of the conserved C-terminal domain. Evidence of expression in piroplasm and sporozoite stages for copies within the *Tpr* locus suggests a functional role in transmission between tick vector and mammalian host.

## Materials and Methods

### *T. parva* DNA Sample Used for PacBio Sequencing

A high molecular weight DNA sample was obtained from *T. parva* piroplasms obtained and preserved in 1992, at the International Livestock Research Institute, from bovine BJ182, which had been experimentally inoculated with the Muguga bulk stabilate 3087. DNA was sheared with the Covaris E210 and Pippin size selection was done to obtain a sample with average fragment length of 17,000 bp.

### PacBio Sequencing and Genome Assembly

Long-read sequencing was performed on the Pacific Biosciences (PacBio) sequencing platform, using the single-molecule, real-time sequencing (SMRT) (Eid et al. 2009)*.* Four SMRT cells were sequenced with C5P3 chemistry (180 min movie), and two additional channels were sequenced at a later time with C6P4 chemistry (240 min movie). The PacBio reads were assembled using the HGAP3 assembler (Chin et al. 2013). Assembly polishing was done with Illumina short reads from the same strain, using the pilon software tool (Walker et al. 2014). Alignment of the reference assembly to the new assembly generated from the PacBio reads was done using NUCmer (v3.1) (Kurtz et al. 2004) and visualized using mplotter (Chakraborty et al. 2018). The gene prediction software Genemark-ES was used to identify ORFs within the closed gaps (Lomsadze et al. 2005) and searches against the *T. parva* reference genome to identify homologs were conducted with BLASTX (Altschul et al. 1990).

### Other Theileria Proteomes Accessed

The predicted proteomes of the following *Theileria* species were used: *Theileria annulata* (Ankara) (Pain et al. 2005), *Theileria orientalis* (Shintoku) (Hayashida et al. 2012), and *Theileria equi* (WA) (Kappmeyer et al. 2012). These proteomes, along with both the *T. parva* reference annotation (Gardner et al. 2005) and re-annotation (Tretina et al. 2020), were accessed from the GenBank repository.

### Theileria Species Tree

To reconstruct the phylogenetic relationship among *Theleria* species, OrthoDB v10.1 was used to identify 1992 groups of orthologous genes that span > 90% species and are present as a single copy in > 90% species in the order Piroplasmida, which includes the *genera Theileria*, *Cytauxzoon* (another Theilerid) and *Babesia*. Ten genes were subsequently randomly selected from the list of orthologous groups provided they are present in *B. bigemina*, *B. bovis, T. annulata, T. equi, T. parva*, and *T. orientalis* in PiroplasmaDB (release 60, 9 Nov. 2022) and the ability to find single copies with BLAST of the genomes of *T. parva lawrencei* (buffalo_3081), *T. parva lawrencei* (Mara6998 c11), *T. parva* Kiambu5, *T. parva* Marikebuni, *T. parva* Uganda, and *Theileria* sp. buffalo N86A. VEuPathDB (Amos et al. 2022) was used to confirm the conserved synteny of these genes across the *Theileria* and *Babesia* species used in the analysis. The ten genes are the following: (a) 26S proteasome subunit 4; (b) 60S ribosomal protein L31; (c) Adenylate kinase 3; (d) Adenylosuccinate lyase; (e) Isocitrate dehydrogenase; (f) Myosin A; (g) Prohibitin; (h) Calcium-dependent protein kinase 4; (i) Ribosomal protein S2; j) Signal recognition particle subunit SRP68. The fasta file with orthologs for each gene was submitted to MAFFT (v7.450) (Katoh and Standley 2013), which chose the L-INS-I alignment algorithm in all cases. The resulting ten alignments were concatenated, and a partition model was passed to RAxML (-q) accounting for each gene, allowing for individual models of nucleotide substitution to be estimated. RAxML's autMRE function determined 250 BS bootstrap (BS) replicates to be adequate and three independent analyses (each with a different starting seed) were run for 750 BS replicates. The resulting maximum likelihood (ML) trees were topologically identical with Robinson Foulds distances of 0.000, as determined by RAxML (v8.2.12) (Stamatakis 2014).

### Multiple Sequence Alignment, Domain Characterization and Phylogenetic Analyses of *T. parva* Muguga Tpr Genes

Multiple sequence alignments of amino acid sequences of all previously identified reference strain *Tpr* genes were conducted using MAFFT, utilizing the E-INS-i algorithm, which is an iterative refinement method designed for multiple conserved domains and recommended if the nature of the sequences to be aligned is unclear (Katoh et al. 2005; Katoh and Toh 2008). To define the conserved region of the aligned *Tpr* gene sequences, Gblocks (v0.91b) was implemented to eliminate poorly aligned positions and divergent regions of the amino acid alignment (Castresana 2000), using options that allowed for smaller final blocks and gap positions (< 10) within the final blocks. The subsequently defined conserved region was used to build a hidden Markov model (HMM) profile using HMMER (v3.3) (Eddy 2011) to search protein sequences of all open reading frames identified in a recent re-annotation of the *T. parva* reference genome (Tretina et al. 2020), and were considered significant if they had a lower HMMER e-value than the highest value obtained from any of the original *Tpr* genes (3.5e-10). The conserved domain of all *Tpr* genes, including those already described, as well as those newly identified by the HMMER search, were aligned with MAFFT. Sequence conservation in the resulting alignment was graphically represented using a sequence logo (Crooks et al. 2004).

To construct a phylogenetic tree with the resulting alignment, ModelTest-NG (v0.1.5) was used to select the best-fit amino acid substitution model (Darriba et al. 2020), which was the PROTGAMMALG model. ML and bootstrapping analyses were conducted with RAxML. ML analysis of the *Tpr* gene sequences employed 1,000 bootstraps, given initial tests using the autoMRE criterion (Pattengale et al. 2010) showed 550 BS replicates to be adequate. Pairwise distances were calculated between the amino acid sequence of the conserved domain of each *Tpr* gene using the p-distance substitution model (Nei and Kumar 2000) in MEGA X (Kumar et al. 2018), applying pairwise deletion to gaps and missing data in the amino acid sequences.

### Tpr Homolog Identification and Genus-Wide Phylogenetic Analysis

The final set of *Tpr* genes in the Muguga strain of *T. parva* was used to build a second amino acid HMM profile to search additional *Theileria* species where whole proteomes are available, using the default HMMER e-value of 1e-03 to determine significant results. Proteomes were readily available for *T. annulata* (Pain et al. 2005), *T. orientalis* (Hayashida et al. 2012), and *T. equi* (Kappmeyer et al. 2012). In addition to the Muguga reference, we included a second cattle-derived *T. parva* strain (Kiambu 5), which was originally described in Morzaria and Williamson (1999). Additionally, with the explicit intent to include *Tpr* genes from buffalo-derived *T. parva*, we included the genome assembly of two *T. parva* genotypes isolated from African Cape buffalo. One isolate, Buffalo_3081, from *T. parva* Lawrencei, was originally described in Morzaria et al. (1995), and its genome assembly was previously made available (Palmateer et al. 2020). A second buffalo-derived *T. parva* genotype was also included here, for isolate Mara6998 (clone 11), which was described previously (Baldwin et al. 1988). Finally, we included isolate N86A, a representative of an undescribed *Theileria* species also found in the African Cape buffalo, named *Theileria* sp. (buffalo) (Allsopp et al. 1993; Bishop et al. 2015). To generate genome assemblies for strains Kiambu 5, Mara6998, and N86A, we applied a whole genome DNA capture method followed by sequencing with an Illumina MiSeq platform, as described before (Palmateer et al. 2020). Sequencing reads were assembled with the SPAdes assembler (v3.9.0) (Nurk et al. 2013). Open reading frames (ORFs) in the queried assemblies were identified using the gene prediction software Genemark-ES (Lomsadze et al. 2005), and translated to amino acid sequences. We searched these sequences using the above described HMM profile, based on the conserved C-terminal domain, to identify likely *Tpr* gene family homologs. ML analysis of the *Tpr* gene evolution in *Theileria* species used the PROTGAMMAVT model and 500 bootstraps were used to construct a phylogenetic tree, given initial tests using the autoMRE criterion showed 400 bootstraps to be adequate. Only the conserved C-terminal domain identified in each translated gene by the HMM search described above were used to construct the phylogenetic tree. The most likely tree from RAxML was rooted on the *T. equi* clade in Dendroscope (v3.7.2) (Huson and Scornavacca 2012). The tree file was imported into iTOL (Letunic and Bork 2021) for rendering radial phylogeny and the coloring was completed in Adobe Illustrator 2021.

## Results

### Data Generation

**Fig. 1:**
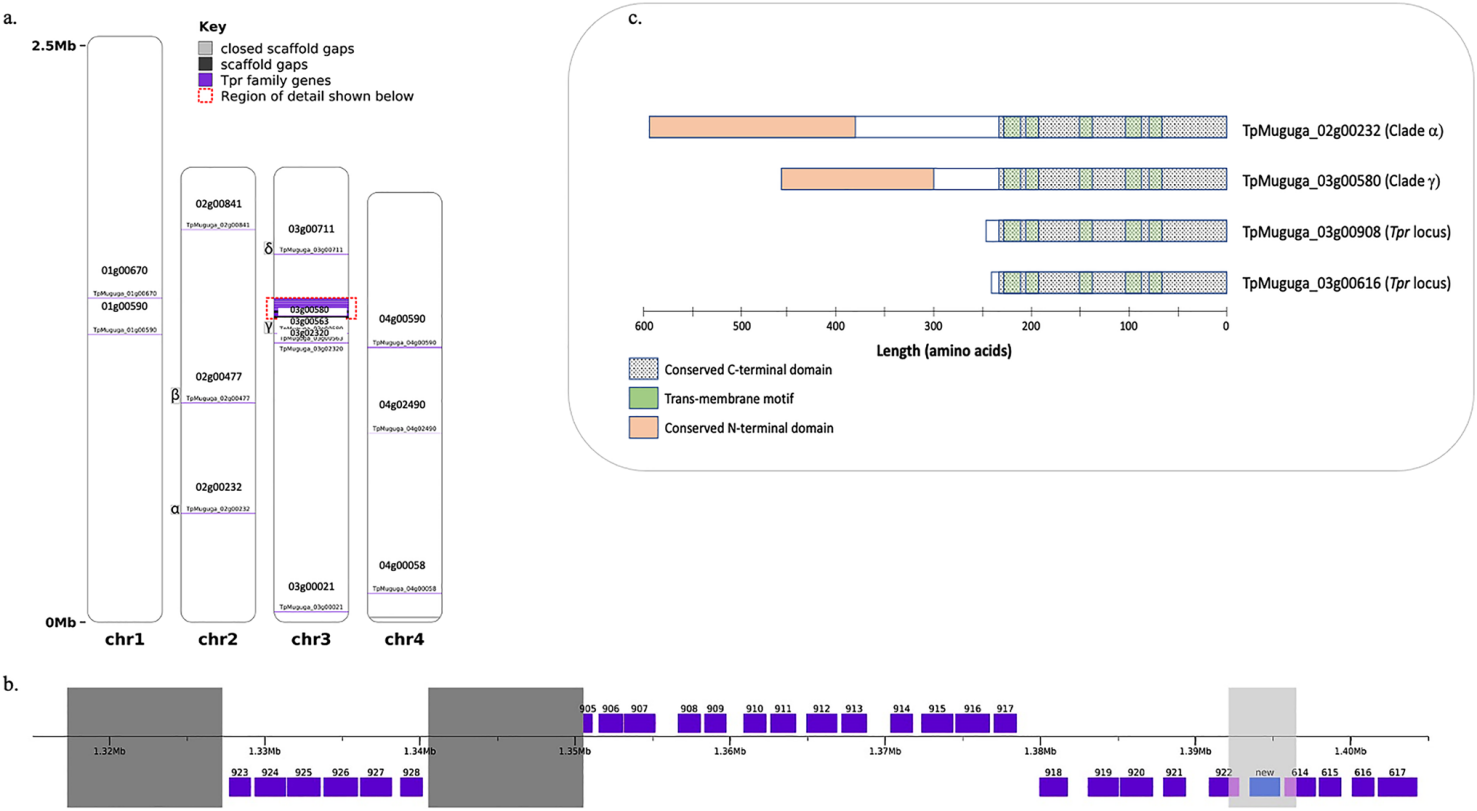
Location of genes encoding Tpr family proteins in the *T. parva* reference genome, and structure of four representative family members. A total of 42 *Tpr* family genes are located throughout the four nuclear chromosomes of *T. parva*, with those outside of the *Tpr* locus shown by the end of their locus tag (**a**), 29 of which are located within a highly repetitive region of chromosome 3, termed *Tpr* locus (**b**). The light gray shaded area indicates the region of chromosome 3 where the assembly gap was closed, allowing for the identification of one additional *Tpr* gene (blue) and completion of two adjacent partial genes (light purple). Each gene block is labelled with the unique portion of the locus tag, the full version of which includes TpMuguga_03g00, followed by the numbers listed. The dark gray shaded areas represent the gaps that still remain in chromosome 3, with the length of each being set at 10 Kb, an approximate estimate. Four genes are labeled with letters of the Greek alphabet, indicating that they are found within clades containing sequences from five or six strains or species, representing highly conserved orthologs, in Fig. 3. A schematic of representative members of the protein family are shown in the inset (**c**), including two Tpr proteins from the *Tpr* locus and the *T. parva* Muguga sequences from clades α and γ

The original genome assembly for the reference *T. parva* Muguga strain contains four gaps, three in chromosome 3 and one in chromosome 4 (Fig. 1). To close these gaps, long-range sequencing was conducted using the PacBio sequencing platform. For the four SMRT cells using C5P3 chemistry, 131,702 (mean 32,925.5 reads per cell) reads were generated, with a mean read length of 4495 bp and a max read length of 31,573 bp. For the two SMRT cells using C6P4 chemistry, 73,951 (mean 36,975.5 reads per cell) reads were generated, with a mean read length of 4744 bp and a max read length of 36,584 bp. Due to the very short mean length of the PacBio sequencing, limitations still exist in our ability to span the gaps in the reference assembly.

### Closing Gaps in the Reference Genome Assembly of *T. parva*

Assembling the PacBio sequence data, using the HGAP3 assembler, resulted in an assembly that is 8,590,058 bp long. This assembly is 240 kb longer than the original genome assembly (as a result of extending several contigs) and resulted in closure of two of the four gaps in the original assembly. The lone gap that remained in chromosome 4, between contigs AAGK01000003 and AAGK01000004 was closed, adding 6996 bp to the length of the *T. parva* genome. The third gap in chromosome 3, between contigs AAGK01000007 and AAGK01000006 was also closed, adding 4373 bp to the genome length (Supplemental Fig. 1).

Within the two PacBio contigs that spanned the gaps in the original reference assembly, ORFs were identified, likely representing new protein-coding genes (Supplemental Table 1). In the now closed chromosome 3 gap, an ORF with a total length of 1961 bp was identified (Fig. 1), which BLAST matched to proteins annotated as “Tpr family”. As this gap was within the *Tpr* locus, this was not unexpected. By closing the gap, we were also able to extend TpMuguga_03g00614, which was truncated in the original assembly, due to its location at the end of the contig adjoining the gap. TpMuguga_03g00614 was previously described as having a length of 1249 bp in the most recent *T. parva* annotation (Tretina et al. 2020), however the length of the ORF we identified containing the truncated gene, along with the additional sequence identified in the gap, totaled 1967 bp. Most importantly, by extending the truncated TpMuguga_03g00614 at the 3′ end, the complete gene sequence now contains the segment encoding the conserved domain present in all *Tpr* genes, allowing us to annotate it as such with greater certainty. We were also able to extend another truncated gene at the opposite end of the gap, TpMuguga_03g00922. This gene had previously been described as 1260 bp long; by extending it at the 5′ end, it is now 1953 bp long. The newly identified *Tpr* gene sequence, along with the updated sequences of the two genes adjacent to the gap, allowed for their use in analysis of the *Tpr* gene evolution in *T. parva* in subsequence sections of the results. Since these are single exon genes, the location of the first in-frame ATG defined the start of each predicted protein. In the newly discovered gene, the first in-frame ATG was 128 codons from the ORF’s 5′ end; in the ORF containing the extended TpMuguga_03g00922, the first in-frame ATG was 129 codons from the ORF’s 5′ end.

**Table 1:** Tpr homologs identified in the predicted proteome of *Theileria* species

Species (strain)	Host	Predominant locations	No. Tpr homologs detected using HMM profile (change from previously reported Tpr-related proteins)
*Theileria parva* (strain: Muguga)	Cattle	Sub-Saharan Africa	42 (+ 3)
*Theileria parva* (strain: Kiambu5)	Cattle	Sub-Saharan Africa	37 (N/A)
*Theileria parva* (strain: Lawrencei)	Buffalo	Sub-Saharan Africa	48 (N/A)
*Theileria parva* (isolate: Mara6998)	Buffalo	Sub-Saharan Africa	54 (N/A)
*Theileria* sp. (buffalo) (isolate: N86A)	Buffalo	Sub-Saharan Africa	35 (N/A)
*Theileria annulata*	Cattle	North Africa & West Asia	101 (+ 8)^a^
*Theileria orientalis*	Cattle	Japan, Australia, New Zealand	22 (+ 17)^b^
*Theileria equi*	Equids	Widespread in tropical and subtropical areas	118 (+ 9)^c^
			Total: 457

^a^Pain et al. (2005), ^b^Hayashida et al. (2012), ^c^Kappmeyer et al. (2012)

In closing the gap in chromosome 4, we identified four new ORFs, using gene prediction software (Supplemental Table 1). A BLASTX search revealed the sequences contained in all four ORFs to be most similar to members of the Sub-telomere-encoded Variable Secreted Protein (*SVSP*) multigene family. This result was also expected, given the location of the gap in the first 20 kb of chromosome 4. The presence of the *SVSP* multigene family at the 5′ end of chromosome 4 (as well as at each end of all four chromosomes) has been described previously (Schmuckli-Maurer et al. 2009).

### Tpr Gene Structure

The first published genome annotation of *T. parva* identified 39 hypothetical proteins containing the conserved C-terminal region that defines a Tpr protein (Gardner et al. 2005). The 2005 annotation was completed using a number of gene finding tools that had been trained using partial or full-length gene sequences from either (i) *T. parva* orthologs of highly conserved eukaryotic genes or (ii) *T. parva* genes expressed in schizont-infected cells that were identified from a full-length cDNA library made from purified schizonts. Protein families were originally identified using TRIBE-MCL, as described in Pain et al. (2005). As methods of gene annotation have improved considerably since then, we sought to refine the definition of the Tpr family protein and identify an updated and comprehensive set of *Tpr* multigene family members in the *T. parva* reference genome.

We first sought to establish the characteristics that define a *Tpr* gene, which then could be used to annotate potential new *Tpr* genes. All genes annotated as *Tpr* in the 2005 annotation shared at least a portion of the common 3′ conserved region, which typically encodes five predicted transmembrane helices (Fig. 1). The high degree of variability in sequence length and lack of sequence similarity in the N-terminal and central regions of many Tpr proteins preclude assessment of conservation across all *Tpr* genes using these regions (but see below for comparison of genes in the *Tpr* locus). The conserved 3′ region, including the presence of five encoded transmembrane domains, was the only shared characteristic across the *Tpr* genes, and was therefore considered the sufficient criterion to determine inclusion into this multicopy gene family. We then verified the current annotation of *Tpr* family ORFs, and sought to identify new ORFs in the *T. parva* genome re-annotation that met this definition. We used a multiple sequence alignment of all previously annotated Tpr family proteins to extracted only the conserved C-terminal domain of the protein, which was 245 amino acids in length (Fig. 1). This conserved block was used to create a hidden Markov model (HMM) profile; this was in turn used to search all translated ORFs throughout the re-annotated *T. parva* genome. As expected, we found support for all 39 protein-coding genes previously annotated as *Tpr* gene family members, since their proteins served as the input to build the HMM profile, and therefore served as a positive control. We also confirmed the inclusion of the protein encoded by the new *Tpr* gene identified in the newly closed gap. Two additional genes met the significance threshold in the HMM search: TpMuguga_02g00232 and TpMuguga_04g02490. TpMuguga_02g00232 was previously annotated as encoding a hypothetical protein and TpMuguga_04g02490 was absent in the original annotation and was identified as a new hypothetical gene during the re-annotation of the *T. parva* genome (Tretina et al. 2020). Of the 42 protein-coding genes identified as being part of the *Tpr* family, 29 are located within the *Tpr* locus of chromosome 3, and 13 are distributed throughout the four nuclear chromosomes of the genome (Fig. 1). Unlike several other notable multigene families such as the SVSP family in *T. parva* and the *var* gene family in *P. falciparum*, that are primarily located in the sub-telomeric regions of the chromosomes (Rubio et al. 1996; Schmuckli-Maurer et al. 2009), the *Tpr* gene family is not sub-telomeric (Gardner et al. 2005).

**Fig. 2:**
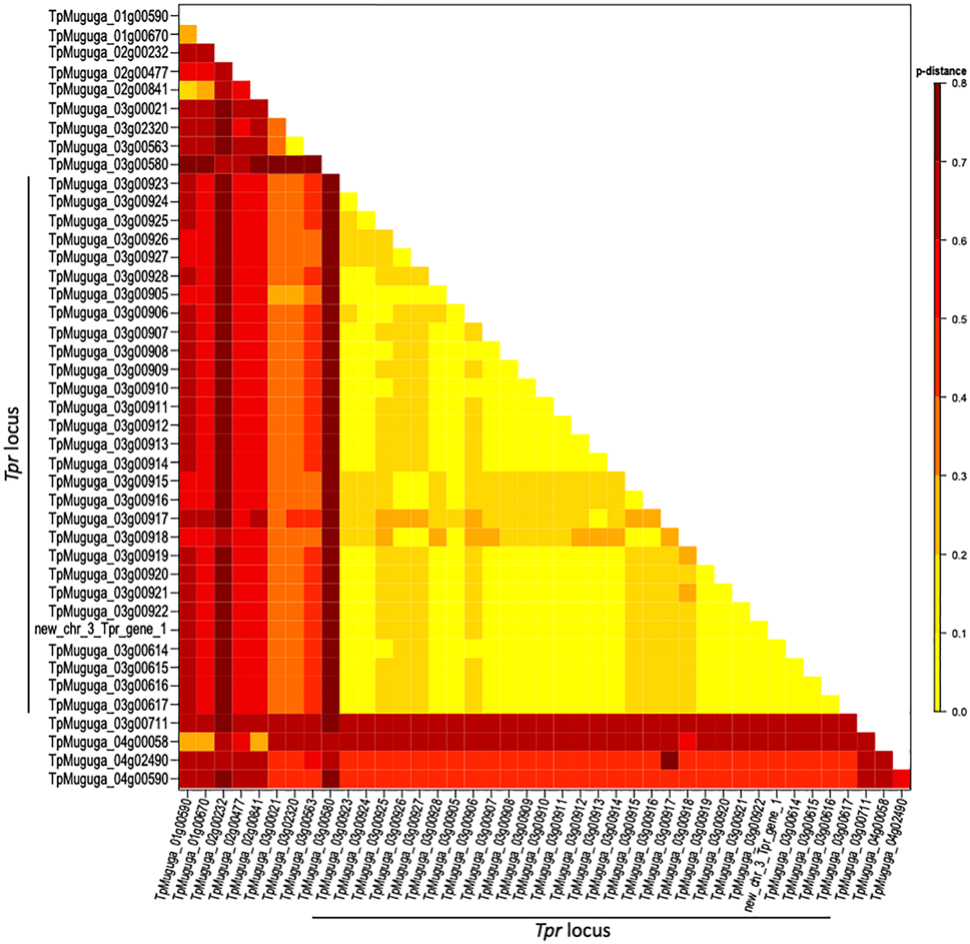
Amino acid pairwise distance comparison of the conserved C-terminal region for all Tpr family proteins in *T. parva*. Pairwise distances were calculated between all known Tpr family proteins in the *T. parva* genome, using only the amino acid sequence encoded by the conserved region at the C-terminus, to make comparisons robust to length variation in the rest of the protein. The *p*-distance substitution model was used, assuming uniform rates of substitution and pairwise deletion to treat gaps and/or missing data. Proteins are ordered based on the position of their encoding gene in the genome, from left to right on the x-axis and top to bottom on the y-axis. Pairwise genetic distances are displayed as a heatmap, from a minimum (yellow) to maximum (dark red) (Color figure online)

Nucleotide sequence comparisons among members of the *Tpr* locus revealed sequence similarity between 76.2 and 100%, an indication of both recent duplications, resulting in identical copies, and older duplication events followed by mutations. Nucleotide sequences of the genes in the *Tpr* locus are too divergent from those in other locations in the genome for reliable alignment, other than in the region encoding the conserved C-terminal domain. The results of a pairwise distance analysis of the conserved C-terminal domain of all Tpr proteins encoded in the Muguga reference genome are displayed as a heatmap (Fig. 2), where proteins are ordered based on the position of their respective encoding genes in the genome. These results confirm that the genes in the *Tpr* locus are most similar to one another; this is shown by the large block of high sequence similarity among the proteins they encode (Fig. 2, in yellow). More specifically, we identified 15 instances where protein sequences were identical in the conserved C-terminal domain, all among those encoded by genes located in the *Tpr* locus. Not unexpectedly, the new gene identified by closing the gap in chromosome 3 between contigs AAGK01000007 and AAGK01000006 was identical in the conserved 3' region to the two *Tpr* genes immediately upstream. Nucleotide similarity to those flanking loci was 100% and 99.06% across the full gene length, likely explaining the difficulty in assembling this genomic region originally, when the assembly was based on Sanger reads (Gardner et al. 2005). On the other extreme are three *Tpr* copies, one chromosome 2 (TpMuguga_02g00232) and two in chromosome 3 (TpMuguga_03g00580 and TpMuguga_03g00711), which are very distinct from most other copies. They show > 60% amino acid sequence divergence from other copies in the conserved C-terminal domain.

Phylogenetic analysis of the conserved domain specific to the Tpr family proteins for all 42 Tpr proteins resulted in two distinct clades reflecting the protein location within the genome (Supplemental Fig. 2). Tpr family proteins encoded by genes within the *Tpr* locus form a single clade, with much shorter branch lengths, reflecting a much higher degree of sequence similarity (Supplemental Fig. 2). This is the result of a shorter evolutionary time between the common ancestor of all these ORFs, of a much stronger functional constraints leading to sequence conservation, or a combination of both factors. The remaining 13 proteins form a separate clade, with longer branch lengths. Similar patterns of sub-grouping have been observed in the *var* multigene family in *P. falciparum* (Lavstsen et al. 2003). It was hypothesized that those *var* genes within each subgroup are more likely to exchange genetic information with each other through gene conversion or unequal crossover, and that the groups may be maintained through inhibition of recombination between heterologous chromosomal locations or different direction of transcription. This may also be the case for the subgroups observed within the *Tpr* gene family.

### Tpr Gene Family Evolution in *Theileria parva* and Closely Related Species

With a definition of the Tpr family protein established, based on the conserved C-terminal domain, we sought to evaluate the level of its conservation in other *Theileria* species.

Using an alignment of this domain found in the larger set of 42 predicted Tpr family proteins (Supplemental Fig. 3a), a second HMM profile was generated to search the publicly available proteome of closely related *Theileria* species: *T. annulata*, *T. equi*, and *T. orientalis*. Our group also recently generated additional whole genome assemblies for *T. parva.* In this study, we included an additional cattle-derived *T. parva* strain, Kiambu 5 and two strains of African Cape buffalo-derived *T. parva*, Lawrencei (buffalo 7014) and Mara6998(cl. 11). Buffalo-derived strains may form a distinct taxon (either at the sub-species or at the species level) from that consisting of cattle-derived *T. parva* strains, including Muguga, on which the reference genome is based (Palmateer et al. 2020). We also generated a whole genome assembly for the closely related, but not yet described, species known to infect the African Cape buffalo, known as *Theileria* sp. (buffalo) (isolate N86A). For these new genome assemblies, ORFs were identified, and their amino acid sequences were searched using the second HMM profile.

**Fig. 3:**
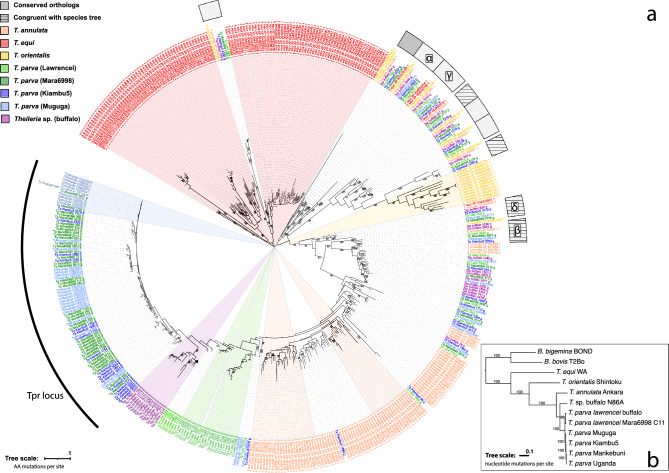
Maximum likelihood phylogenetic tree of all *Theileria* Tpr family protein sequences identified with an HMM profile derived from the conserved domain using all *T. parva* homologs. Monophyletic clades where all sequences are from a single taxon and at least eight sequences are present, are colored by species, with a wedge (wedges label only the sequences, i.e., terminal nodes, and not to internal nodes). Monophyletic clades containing sequences from five or six strains or species, representing highly conserved orthologs, are denoted by solid gray boxes outside the tree. These monophyletic clades containing a Muguga Tpr homolog in are marked with a letter of the Greek alphabet, with their corresponding location in the genome marked in Fig. 1. Monophyletic clades containing sequences from three or four species, representing clades congruent with the species tree, but result from gene loss or duplication, are denoted by hatched gray boxes outside the tree. Percent bootstrap support, based on 500 replicates, is shown for nodes with greater than 70% support. **b** Maximum likelihood phylogenetic tree of one of three topologically identical trees for 10 single copy genes. Percent bootstrap support, based on 750 replicates, is shown for nodes with greater than 70% support

Genes encoding Tpr family protein homologs were identified in the *Theileria* genomes examined (Table 1). For the three *Theileria* species where *Tpr*-related genes had been previously identified, our search yielded a larger number of undescribed homologs in each case: an increase of eight in *T. annulata*, 17 in *T. orientalis*, and nine in *T. equi*. We also identified 48 and 54 *Tpr* family homologs in the buffalo-derived strains of *T. parva* Lawrencei and Mara6998, respectively. This represents an increase in the *Tpr* gene count found in *T. parva* Muguga, with an additional six genes in *T. parva* Lawrencei and 12 genes in Mara6998. This is likely an underestimate of the total number of *Tpr* family genes present in the buffalo-derived strains of *T. parva* given their fragmented genome assemblies. These genomes were assembled using 250 bp long Illumina reads, and there are > 100 gaps in each assembly, where additional copies may lie. This is a situation similar (but perhaps more drastic) to that encountered during the assembly of the original genome of reference Muguga strain of *T. parva* (Gardner et al. 2005). There were 35 copies of *Tpr* gene family homologs identified in *Theileria* sp. (buffalo), contradicting previous PCR results suggesting their absence (Pienaar et al. 2011). Again, this is likely an underestimate, given the issue of a fragmented assembly.

To determine if any of the *Tpr* genes has orthologs across *Theileria* species, we looked for the presence of clades of *Tpr-*encoded proteins and their homologs in other species for which the phylogenetic relationship is congruent with the species tree. The species used in this study were previously included in phylogenetic studies (Lack et al. 2012; Sivakumar et al. 2014). However, the resolution of the triad (*T. parva*, *T. annulata*, *Theileria* sp. (buffalo)) is incongruent between studies and any resolution lacks bootstrap support because the locus used (18S rDNA) evolves too slowly to inform the order of speciation of these closely related taxa. Therefore, here, we reconstructed the phylogenetic tree of these taxa by maximum likelihood, using the concatenation of ten single copy loci present in the Piroplasmida (Methods; Supplemental Fig. 4). The analyses support a tree in which *Theileria* sp. (Buffalo) is sister *T. parva*, and *T. annulata* is an outgoup to those taxa (Fig. 3).

A maximum likelihood analysis of proteins encoded by the *Tpr* gene homologs found in the eight genomes of the six *Theileria* species/lineages analyzed yielded a phylogenetic tree with several remarkable findings (Fig. 3). The presence of Tpr homologs across the genus marks this gene family as an ancient genomic feature. It is important to note that many of the relationships in this tree are poorly resolved, due to very short internal branches, and only bootstrap values above 70% are shown. Nevertheless, the phylogenetic relationships across elements of different species revealed a wide repertoire of modalities of evolution, including (i) preservation of orthologous copies, (ii) gene loss, (iii) gene duplication, and (iv) both old and recent rapid species-specific gene family expansions.

There were several instances of monophyletic clades containing sequences that follow the same pattern of relationship between the *Tpr* orthologs as the species from which they were obtained. Some clades contained orthologous genes from all, or nearly all (at least five of the six) *Theileria* taxa (e.g., clades marked with solid gray boxes outside the phylogenetic tree in Fig. 3). These cases of clades within the Tpr tree that conformed to the species tree suggest strict transmission of orthologs, without duplications or losses. These genes are located in chromosomes 2 and 3 in Muguga, with the clades containing *Tpr* gene copies found in Muguga denoted by letters of the Greek alphabet in Fig. 3, and their corresponding spot similarly indicated in Fig. 1. Interestingly, the three copies with the most distinct sequence in the *T. parva* reference strain (Muguga) belong to three of these clades, α, δ and γ. In all three cases, the ortholog group defined by the phylogenetic analysis is syntenic in the genomes of *T. parva*, *T. annulata* and *T. equi*, but not in *T. orientalis*, *B. bovis* or *B. microti*, as shown in VEuPathDB (Amos et al. 2022). In addition, the orthologs for clades δ and γ are also syntenic in *Cytauxzoon felis*, a genus closely related to *Theileria*.

Clades in which the *Tpr* gene tree conforms with the species tree, but where only three or four *Theileria* species or *T. parva* subpopulations from cattle and buffalo are present, suggest either gene loss or genes missed in incomplete assemblies; examples include clades marked with hatched gray boxes outside the phylogenetic tree in Fig. 3, two of which do not have representatives in *T. parva* Muguga but are present in other *T. parva* isolates, such as Kiambu5, suggesting a recent deletion in the reference Muguga strain.

A number of species-specific expansions of *Tpr* genes were identified, defined arbitrarily by the presence of at least eight sequences from the same species in a single monophyletic clade (Tpr sequences marked with colored wedges in Fig. 3, and well as the large *Tpr* locus in *T. parva*). In some instances, they appeared to be the result of gradual expansions (inferred, somewhat tentatively, from long internal and terminal branches, as seen in *T. orientalis*—Fig. 3, Tpr sequences in yellow wedge); in others, rapid but old expansions (suggested from very short internal branches and long terminal branches, as seen in one monophyletic clade containing *T. equi*—Fig. 3, Tpr copies in red wedge furthest to the right); and a third case represents rapid but relatively recent expansions and/or gene conversions events, characterized by large groups of nearly identical sequences—most dramatically represented by the sequences from the *Tpr* locus in the Muguga, Kiambu 5, and Mara6998 strains of *T. parva* (labeled in Fig. 3), but also in *T. parva* Lawrencei (from buffalo) (Fig. 3, Tpr copies in light green wedge). Interestingly, the *Tpr* locus in *T. parva*, consisting of a cluster at least 29 genes, includes blocks of genes encoded in opposite strands (Fig. 1), consistent with both simple gene duplication as well as segmental duplication coupled with inversion.

While some of these specific cases could be altered by inclusion of possible missed copies from incomplete genomes or additional sampling of other strains or species, the fact that the phylogeny includes substantial repertoires of *Tpr* genes from several genomes suggests that all these modes of evolution occurred at some point or another in one or more species.

### Conserved Domains in Orthologous Gene Copies

In instances of orthologous copies preserved across species (clades labeled with gray boxes in Fig. 3), we sought to identify any additional domains across the full-length protein sequence, outside of the conserved C-terminal domain. There were two such monophyletic clades that contained all six taxa: the clades containing two highly distinct Tpr copies, encoded by TpMuguga_02g00232 (the copy closest to the 3′ end of chromosome 2) and by TpMuguga_03g00580 (the copy outside of, but closest to, the *Tpr* locus, in chromosome 3). After aligning the six sequences in each clade, a conserved block was identified specific to each alignment (Fig. 1c; Supplemental Fig. 5), using the same methods as described in identifying the previously mentioned conserved block found in all *Tpr* genes. This resulted in the identification of new conserved blocks of 213 and 145 amino acids in the orthologous groups containing TpMuguga_02g00232 (clade α) and TpMuguga_03g00580 (clade γ), respectively.

A BLAST search with the 213 amino acid-long conserved block from TpMuguga_02g00232 yielded 189 hits (e-value ≤ 1e-5) against NCBI’s non-redundant protein database, 181 of which were to the sequences derived from the genus *Theileria*, two to a gene product annotated as “Tpr related protein, putative” in *Babesia microti* (strain RI), and six hits to a gene with product annotated as “cf63 antigen” in *Cytauxzoon felis*. Neither is in a syntenic genomic position relative to *T. parva*. In both of those species, the homologs contain multiple transmembrane motifs (11 predicted in the *B. microti* protein and 12 in the *C. felis* protein), suggesting that both proteins are membrane-bound. A BLAST search of the conserved 145 amino acid-long block in TpMuguga_03g00580 yielded similar results, with 193 total hits (e-value ≤ 1e-5), 183 of which to *Theileria* species, four to the “Tpr related protein, putative” genes in *B. microti* (strain RI), and six hits to a gene annotated as “cf63 antigen” in *C. felis*. The *C. felis* gene is in a syntenic genomic location as its ortholog in *T. parva*. The cf63 antigen has previously been identified as a potential vaccine candidate against cytauxzoonosis in domestic cats, based on a high level of conservation and expression in the schizont stage of the life cycle; however, the function of this gene is unknown (Khana et al. 2018). Another interesting result of this BLAST search was that while the conserved region was found in 5–12 genes in *T. parva*, *T. annulata*, and *T. orientalis* (the cattle-infecting *Theileria* species), it matched to 63 genes in *T. equi*, suggesting a more substantive role in that species. These 63 matches all fell within the monophyletic clade of *T. equi* in Fig. 3. However, it should be noted that the *T. equi* genome is approximately 3 Mbp larger than that of *T. parva* and *T. annulata*. It could therefore be a more general property of the genome without implications for function.

## Discussion

In the course of this study, we closed two of the remaining four gaps in the reference genome assembly of *T. parva*, one of three in chromosome 3 and the remaining gap in chromosome 4. The latter resulted in three of the four nuclear chromosomes now comprising a single supercontig. This was facilitated by the application of a long-read sequencing platform, specifically PacBio, which allowed us to accomplish what could not be done with Sanger reads, which are shorter in length than Tpr- or SVSP-encoding genes. Two gaps in chromosome 3 could not be closed, likely as a result of unusually short PacBio read lengths, resulting from a relatively degraded genomic DNA sample. A complete nuclear genome assembly, when completed, will likely result in the identification of additional *Tpr* family members identical to those that flank them. In the closed gap of chromosome 3, we identified one new complete *Tpr* gene, which is identical in sequence to one of its flanking genes, as well as the ends of two previously truncated *Tpr* genes.

The work reported here represents the most thorough characterization to date of the *Tpr* gene family in *T. parva*, and its homologs in closely related *Theileria* species. By utilizing a re-annotated genome (Tretina et al. 2020) and summarizing what has been learned about the *Tpr* gene family in the 17 years since it was first characterized in the reference genome (Gardner et al. 2005), we were able to identify additional gene family members across the *T. parva* genome and evaluate their level of sequence similarity. To further strengthen our understanding of the evolution of the gene family, we evaluated homologs in the genome of other *Theileria* species which, except for *T. annulata*, were not available in 2005, when these genomes were first published.

Our work shows the presence of three broad classes of *Tpr* genes: (i) those generated through large species-specific gene family expansions; (ii) a few, isolated copies with syntenic orthologs in other *Theileria* species; (iii) and some are only “semi-conserved”, resulting from a few taxon/strain-specific duplications, with similar copies in closely related strains/species. Evidence from gene expression studies, discussed below, suggests that these classes may be associated with different functions.

While it was known that *Tpr* genes are hyper-polymorphic between *T. parva* isolates (Allsopp et al. 1989; Bishop et al. 1997, 1993; Conrad et al. 1987), our results show that those within the *Tpr* locus in the *T. parva* reference strain have a very high degree of sequence similarity. This may be an example of a series of rapid gene duplications that presented a quantitative advantage through increased protein production (Assogba et al. 2018); conversely, copy number amplification may not be advantageous for the organism, but mutation and selection have not yet had time to act. However, the observation that genes nearly identical to those in the *Tpr* locus array are present in the genomes of multiple *T. parva* isolates from both cattle and buffalo, combined with the large, recent expansion in copy number, and the piroplasm stage-specific expression of genes within the array (Bishop et al. 1997), is consistent with a *Tpr* locus function in *T. parva* distinct from those of other copies in the genome.

Recent gene expression analyses applied to different stages of the *T. parva* life cycle (Atchou et al. 2020; Tonui et al. 2018) may help offer insight into the function of putative *Tpr*-encoded proteins. Atchou et al. (2020) sought to identify *T. parva* genes that are expressed in a stage-specific manner within the mammalian host through a differential gene expression study in schizonts (when the parasite is present in the mammalian lymphocytes) and piroplasms (parasite in red blood cells, prior to transmission to the tick vector). Of 116 genes preferentially expressed in the piroplasm stage, 20 are *Tpr* family members and 19 of these are from the *Tpr* locus. This finding is consistent with earlier results in which piroplasm transcripts matched most closely to sequences within the *Tpr* locus (Bishop et al. 2005). Tonui et al. (2018) examined transcript levels in the sporoblast, sporozoite, and schizont stages, and found 22 of the 41 *Tpr* genes with detectable transcripts. Of the 22, 13 were located within the *Tpr* locus, and all of these genes demonstrated the highest expression levels in the sporozoite stage, the stage that infects the mammalian host. In summary, *Tpr* genes from the *Tpr* locus appear to be preferentially expressed in the piroplasm stage (*n* = 16) or the sporozoite stage (*n* = 13), with nine genes present in both sets. The preferential expression of *Tpr* locus family members in the piroplasm and sporozoite stages of the life cycle suggests that the *Tpr* locus-encoded proteins play a specific role during entry into a new host, be it the entry of sporozoites into mammalian leukocytes or the establishment of piroplasm infection of the mammalian red blood cells (or in preparation for transfer to the tick). The rapid, independent, species-specific family expansions, and the high *d*_N_/*d*_S_ (a measure of selective constraint on the encoded protein) (Weir et al. 2010), are all suggestive of rapid evolution, consistent with an “arms race-like” evolution often seen in molecules involved in host-parasite interactions. Together with the presence of Tpr family proteins as integral membrane proteins, based on the transmembrane helices at the conserved C-terminal domain (Bishop et al. 1997), these observations are consistent with a role in host/vector cell binding.

While genes within the *Tpr* locus appear to have few detectable transcripts in the schizont stage, Tonui et al. (2018) also found that the *Tpr* genes outside the tandemly arrayed *Tpr* locus had similar transcripts levels across all three life cycle stages examined. This pattern is consistent with distinct functions for the tandemly arrayed and dispersed *Tpr* loci in *T. parva*. Vertebrate host-parasite-tick co-evolution has been considered in other apicomplexan genera (Jalovecka et al. 2019). Since the conserved C-terminal domain appears to anchor the protein presumably within the parasite cell membrane, the tremendous sequence diversity in the rest of the protein raises the possibility that one of the roles of some of these proteins may be to serve as an immune system decoy, as is presumed to be the case for hypervariable domains within the *var* genes in *P. falciparum* (Diez et al. 2009).

A recent study of *T. annulata* identified 87 genes associated with life cycle transitions, 14 of which were genes that we identified as *Tar*—or *Tpr* gene family homologs in *T. annulata*, using our HMM based on the C-terminal protein domain (Cheeseman et al. 2021). Of these 14, ten appeared to be species-specific, based on Fig. 3, while only four (XP_955068, XP_953969, XP_952979 and XP_953755, with locus tags TA04240, TA05770, TA07765, and TA16870), forming a monophyletic clade on the tree, were semi-conserved across *T. annulata* and *T. parva* strains. This further suggests that the species-specific *Tpr*-like copies (much like the ones composing the *Tpr* locus in *T. parva*) play a distinct role from the more highly conserved *Tpr* genes.

Evolutionarily recent expansions of *Tpr* homologs were observed in other *Theileria* species, containing varying numbers of *Tpr*-like genes, similar to what has been observed between different isolates of *T. parva* (Bishop et al. 1997; Weir et al. 2009). These observations, coupled with the different modes of evolution observed, including ortholog conservation in some instances, gene loss and gene duplication, as well as the presence of several potential pseudogenes (based on the lack of a start methionine codon), suggest that the *Tpr* gene family evolves according to a classic birth-and-death model (Nei and Rooney 2005) coupled with the sporadic co-option of copies into specific functions, which then may lead to ortholog retention. This is consistent with a role in the generation of evolutionary novelty, as suggested before, based on the hypervariability of 5′ ends of selected gene copies, to the extent that the predicted proteins appear to lack detectable N-terminal sequence similarity when different *T. parva* genotypes are compared (Bishop et al. 1997). This hypervariability likely plays an important role in parasite adaptation and/or survival in a hostile host environment.

While the major focus of this study was directed towards the conserved domain in the C-terminal end of the Tpr family protein, which represents the only region present in all homologs that could be consistently aligned, it has been observed that the 5′ end of these genes demonstrates a tremendous amount of variability (Bishop et al. 1997). The ratio *d*_N_/*d*_S_ among all *Tpr*/*Tar* genes in *T. parva* and *T. annulata* was observed to be higher than in any other multigene family with shared homologs in the two species (Weir et al. 2010). Given the high level of conservation we found within the conserved 3′ region, and the fact that the 5′ is sometimes missing and often impossible to align reliably, our genome wide analysis confirms that most of the genetic variation is accumulating in the 5′ prime end of the gene, as initially indicated by Southern blot and limited sequence data from different isolates (Bishop et al. 1997).

A search of the predicted proteomes from *Babesia bovis* and *B. microti* with the HMM profile built from all C-terminal conserved motifs in *T. parva*’s Tpr proteins revealed only seven hits in the former and three in the latter species, suggesting that either *Tpr*-like homologs existed as a low copy gene family in the ancestor of the Piroplasmids and later expanded into large multigene families in some lineages, or that copies were lost through deletion or mutation in *Babesia* species. Interestingly, at least two of these copies are old on an evolutionary timescale, as suggested by the presence of syntenic orthologs between *Theileria* and *Cytauxzoon*, as well as non-syntenic homologs in *B. microti*. The genera *Cytauxzoon* and *Babesia* are distantly related to *T. parva*, separated from it likely by tens of millions of years (Knowles et al. 2018; Lack et al. 2012). Overall, these results show that the origin of antigenic sequences encoded by some of the *Tpr* genes predate the origin of the family Theileriidae and possibly even the origin of the order Piroplasmida. However, subsequently, taxon-specific evolution took place, with some of the oldest copies possibly retaining ancestral functions, but with the vast majority of copies resulting from taxon-specific expansion.

Two of the three genes with the highest transcript levels in the schizont stage were TpMuguga_02g00232 and TpMuguga_03g00580, the two genes from clades α and γ that were conserved throughout all six *Theileria* taxa evaluated in this study, and contained conserved segments in the 5′ region, as well as the 3′ region of the gene (Supplemental Fig. 4). The suggestion by Khana et al. (2018) that the cf63 antigen in *C. felis*, which contains a segment with significant sequence similarity to this conserved 5′ domain, be considered as a vaccine candidate against disease caused by *C. felis* infection in cats, brings up the interesting possibility that these two genes in *T. parva* may be worthy of further study as vaccine candidates.

By closing two of the remaining gaps in the *T. parva* reference assembly, we report several additional multigene family members, including in the *Tpr* gene family. While the specific function of the *Tpr* gene remains unknown, the data presented in this study provide potential clues. As a result of our identification and analysis of the conserved domains in the *Tpr* gene in *T. parva*, future research on this multigene family can be done in the contexts of genome location and inter-species sequence conservation, which may prove valuable. Key objectives that have so far remained elusive include conducting experimental studies to validate the presence and localization of the predicted proteins, and the fitness impact of specific copy knockouts.

### Supplementary Information

Below is the link to the electronic supplementary material.

Supplementary file1 (DOCX 2498 kb)

## Data Availability

Raw sequence reads are available from NCBI, under the following BioProject accession numbers: PRJNA656576 (*T. parva*—Muguga (SAMN15793554)). PRJNA744557(*T. parva parva*—Kiambu 5 (SAMN20526559) and Muguga_BJ182 (SAMN20526561)). PRJNA656583 (*T. parva lawrencei*—Lawrencei (SAMN15466804)). PRJNA744561 (*T. parva lawrencei*—Mara_6998_C11 (SAMN20526560). PRJNA744563 (*T.* sp. (buffalo)—N86_A (SAMN20526558).
