# Supplementary material for: The Hypervariable Tpr Multigene Family of Theileria Parasites, Defined by a Conserved, Membrane-Associated, C-Terminal Domain, Includes Several Copies with Defined Orthology Between Species
Source: J Mol Evol. 2023 Nov 28;91(6):897–911. doi: 10.1007/s00239-023-10142-z (PMC10730637; doi:10.1007/s00239-023-10142-z)
Supplement: Supplementary file 1 — Supplementary file1 (DOCX 2498 kb) [file 239_2023_10142_MOESM1_ESM.docx]

**The hypervariable *Tpr* multigene family of *Theileria* parasites, defined by a conserved, membrane-associated, C-terminal region, includes several copies with defined orthology between species**

Journal of Molecular Evolution

Nicholas C. Palmateer^1^, James B. Munro^1^, Sushma Nagaraj^1^, Jonathan Crabtree^1^, Roger Pelle^2^, Luke Tallon^1^, Vish Nene^2^, Richard Bishop^3^, Joana C. Silva^1,4§^

**Supplemental Figures**


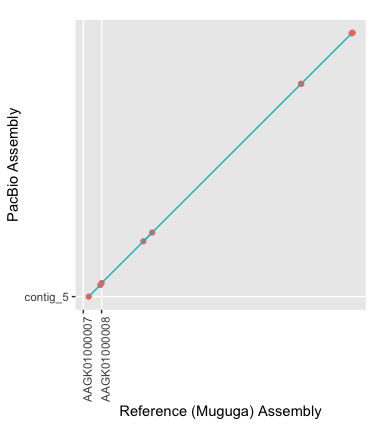

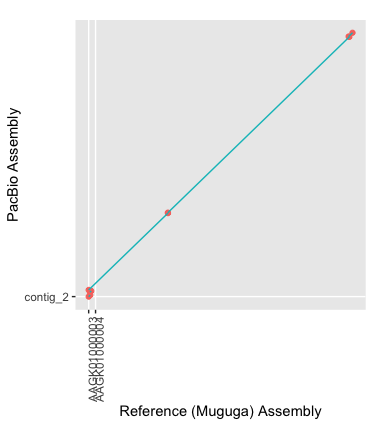


a.

b.

**Supplemental Figure 1: Alignment of contigs demonstrating closed gaps in the *T. parva* Muguga reference, using assembly generated from PacBio sequencing.**

Contigs from the PacBio assembly that span the gaps that exist in the *T. parva* reference assembly are shown aligned to the corresponding two contigs in the reference assembly. The blue lines indicate the segments in alignment from the contigs in each assembly, and the red dots indicate the ends of the assembly contigs. Contig 5 in the PacBio assembly is shown aligned to contigs AAGK01000007 and AAGK01000008 in chromosome 3 (a), and totals 600,141 bp in length. Contig 2 of the PacBio assembly is aligned to the corresponding regions of AAGK01000003 and AAGK01000004 in chromosome 4 (b), and totals 1,895,101 bp in length.

Supplemental Figure 2: Phylogenetic analysis of the conserved C-terminal region among the proteins encoded by the final set of *Tpr* genes found in *T. parva*.

The phylogenetic tree, based on the amino acid sequences of the conserved C-terminal region of Tpr proteins, shows the relationship of the 39 previously identified Tpr proteins, the proteins encoded by the two genes newly annotated as *Tpr* and by the one *Tpr* gene identified as a result of closing a gap in chromosome 3. The tree is rooted using a Tpr*-*related protein family member found in *T. annulata* (XP_953862.1), which was used as an outgroup. Bootstrap values for 1,000 replicates are shown for nodes with greater than 70% support.

**a.**

TpMuguga_03g00614 PFLMVLVGMGLVYCIYPAIAPGMIVPFYLIDKIEMVLLIMTIFPALYVAIARSGKLIPGF

TpMuguga_03g00910 PFLMVLVGMGLVYCIYPAIAPGMIVPFYLIDKIEMVLLIMTIFPALYVAIARSGKLIPGF

TpMuguga_03g00615 PFLMVLIGMGLVYCIYPAIAPGMIVPFYLVDKIEMVLLIATIFPALYVAIARSGKLIPGF

TpMuguga_03g00920 PFLMVLIGMGLVYCIYPAIAPGMIVPFYLVDKIEMVLLIATIFPALYVAIARSGKLIPGF

TpMuguga_03g00617 PFLMVLIGMGLVYCIYPAIAPGMIVPFYLVDKIEMVLLIATIFPALYVAIARSGKLIPGF

TpMuguga_03g00905 ------------------------------------------------------------

TpMuguga_03g00907 PFLMVLVGMGLVFCIYPAIAPGMIVPFYLIDKIEMVLLIATIFPALYVAIARSGKLIQGF

TpMuguga_03g00922 PFLMVLVGMGLVYCIYPAIAPGMIVPFYLIDKIEMVLLIATIFPALYVAIARSGKLIPGF

new_Tpr_chr_3_gap PFLMVLVGMGLVYCIYPAIAPGMIVPFYLIDKIEMVLLIATIFPALYVAIARSGKLIPGF

TpMuguga_03g00921 PFLMVLVGMGLVYCIYPAIAPGMIVPFYLIDKIEMVLLIATIFPALYVAIARSGKLIPGF

TpMuguga_03g00909 PFLMVLVGMGLVYCIYPAIAPGMIVPFYLVDKIEMVLLIATIFPALYVAIARSGKLIPGF

TpMuguga_03g00923 PFLMVLVGMGLVYCIYPAIAPGMIVPFYLVDKIEMVLLIATIFPALYVAIARSGKLIPGF

TpMuguga_03g00911 PFLMVLVGMGLVYCIYPAIAPGMIVPFYLVDKIEMVLLIATIFPALYVAIARSGKLIPGF

TpMuguga_03g00912 PFLMVLVGMGLVYCIYPAIAPGMIVPFYLIDKIEMVLLIATIFPALYVAIARSGKLIQGF

TpMuguga_03g00919 PFLMVLVGMGLVFCIYPAIAPGMIVPFYLVDKIEMVLLIATIFPALYVAIARSGKLIPGF

TpMuguga_03g00924 PFLMVLVGMGLVFCIYPAIAPGMIVPFYLIDKIEMVLLIATIFPALYVAIARSGKLIQGF

TpMuguga_03g00914 PFLMVLVGMGLVFCIYPAIAPGMIVPFYLIDKIEMVLLIITIFPALYVAIARSGKLIQGF

TpMuguga_03g00928 PFLMVLVGMGLVFCIYPAIAPGMIVPFYLIDKIEMVLLIITIFPALYVAIARSGKLIQGF

TpMuguga_03g00913 PFLMVLVGMGLVFCIYPAIAPGMIVPFYLVDKIEMVLLIMTIFPALYVAIARSGKLIQGF

TpMuguga_03g00616 PFLMVLVGMGLVYCIYPAIAPGMIVPFYLVDKIEMVLLIATIFPALYVAIARSGKLIPGF

TpMuguga_03g00908 PFLMVLVGMGLVYCIYPAIAPGMIVPFYLIDKIEMVLLIATIFPALYVAIARSGKLIPGF

TpMuguga_03g00916 PFLMVLVGMGLVYCIYPAIAPGMIVPFYLVDKIEMVLLIATIFPPLIVAIVTKYK----F

TpMuguga_03g00915 PFLMVLVGMGLVYCIYPAIAPGMIVPFYLVDKIEMVLLIATIFPPLIVAIVTKYK----F

TpMuguga_03g00927 PFLMVLVGMGLVYCIYPAIAPGMIVPFYLVDKIEMVLLIATIFPPLIVAIVTKYK----F

TpMuguga_03g00926 PFLMVLVGMGLVYCIYPAIAPGMIVPFYLVDKIEMVLLIATIFPPLIVAIVTKYK----F

TpMuguga_03g00906 PFLMVLVGMGLVYCIYPAIAPGMIVPFYLIDKIEMVLLIATIFPALYVAIARSGKLIPGF

TpMuguga_03g00925 PFLMVLVGMGLVYCIYPAIAPGMIVPFYLIDKIEMVLLIATIFPALYVAIARSGKLIPGF

TpMuguga_03g00917 PFLMVLVGMGLVYCIYPAIAPGMIVPFYLVDKIEMVLLILTIFPALYVAVARSGKLIQGF

TpMuguga_03g00918 PFLMVLVGMGLVYCIYPAIAPGMIVPFYLVDKIEMVLLIATIFPPLIVAIVTKYK----F

TpMuguga_03g00021 PVIMVLVGMGVVYAVYPAIAPGMIVPFYLIDKIEMVLLILTCFPPVIVAVVSREKY----

TpMuguga_03g02320 PVLMIIVGMGLVYMVYPAIAPGLIVPFYLVDKIEMVLLIATFFPPVIQAVLIKHK-----

TpMuguga_03g00563 PVLMIIVGMGLVYMVYPAIAPGMIVPFYLVDKIEMFILIASIVPPVIQAILIKHK-----

TpMuguga_04g02490 ------------------------------------------------------------

TpMuguga_04g00590 PVLMVIVGMGLVYAIYPNIVPGLIGSEYLIDRIAMIIMLFAPVPIAIIATLKHLKIGD--

TpMuguga_01g00590 AMVMCVFAQGVVYVFYPAIAPGMLVDFRNVTKIDQALLIIAPIPYITIAILDVNEETK--

TpMuguga_02g00841 PMIMCIFAQGVVYVFYPAIAPGLLVDFRHVNKIDQALLIMAPIPYITIAILDATNETK--

TpMuguga_04g00058 PVTMCIFAQGVVYVFYPAIAPGLLVDFRHVTKIDQALLIIAPIPSITFAILDATVNA---

TpMuguga_01g00670 AMVMCVFAQGVVYVFYPAIAPGLLVDFRHVNKIDQALLVIAPIPSITSALLSKFYD----

TpMuguga_02g00477 PILMTSVALSLIYAYYPGIAPGLLVDFRYVHKIDLVLMFIVPLPSIVIAVLSYS------

TpMuguga_03g00711 GMLMCIIGLGIIYAVYPGIAPGQIMDFENLQRLEMINLIVSALPSLIIALISEVT-----

TpMuguga_03g00580 PILITFFGLGYVYIVYPAIAPYKLTELRPAYRIDLVCVFAVGLASTIIVFLCEYT-----

TpMuguga_02g00232 PILMSTIAMGLVYFVFPAIAPYRFTDLVTAHRIDVSILITFIAPAVINIILCGY------

**Supplemental Figure 3a, continued**

TpMuguga_03g00614 GGFFNPVSPTCNWGAKN-------ELPWIPGV---PLGQGYHWHLTDLVIPTMIILAYLF

TpMuguga_03g00910 GGFFNPVSPTCNWGAKN-------ELPWIPGV---PLGQGYHWHLTDLVIPTMIILAYLF

TpMuguga_03g00615 GGFFNPVSPTCNWGAKN-------ELPWIPGV---PLGQGYHWHLTDLVIPTMIILAYLF

TpMuguga_03g00920 GDFFDPVSPTCNWGAKN-------ELPWIPGV---PLGQGYHWHLTDLVIPTMIILAYLF

TpMuguga_03g00617 GGFFNPVSPTCNWGAKN-------ELPWIPGV---PLGQGYHWHLTDLVIPTMIILAYLF

TpMuguga_03g00905 ----------------------------------------------------MIILAYLF

TpMuguga_03g00907 GGFFDPVSPTCNWGATN-------KQPWNPGV---NLGQGYHWHLTDLVIPTMIILAYLF

TpMuguga_03g00922 GGFFDPVSPTCNWGATN-------KPVWLPS-----LGTGYHWHLTDLVIPTMIILAYLF

new_Tpr_chr_3_gap GGFFDPVSPTCNWGATN-------KPVWLPS-----LGTGYHWHLTDLVIPTMIILAYLF

TpMuguga_03g00921 GGFFDPVSPTCNWGATN-------KPVWLPS----DLGTGYHWHLTDLVIPTMIILAYLF

TpMuguga_03g00909 GGFFDPVSPTCNWGATN-------KPVWLPS----DLGTGYHWHLTDLVIPTMIILAYLF

TpMuguga_03g00923 GGFFDPVSPTCNWGATN-------KPVWLPS----DLGTGYHWHLTDLVIPTMIILAYLF

TpMuguga_03g00911 GGFFDPVSPTCNWGATN-------KPVWLPS----DLGTGYHWHLTDLVIPTMIILAYLF

TpMuguga_03g00912 GGFFDPVSPTCNWGATN-------KPVWLPS----DLGTGYHWHLTDLVIPTMIILAYLF

TpMuguga_03g00919 GGFFDPVSPTCNWGATN-------KPVWLPS----DLGTGYHWHLTDLVIPTMIILAYLF

TpMuguga_03g00924 GGFFNPVSPTCNWGAKN-------ELPWIPGV---PLGQGYHWHLTDLVIPTMIILAYLF

TpMuguga_03g00914 GGFFNPVSPTCNWGAKN-------ELPWIPGV---PLGQGYHWHLTDLVIPTMIILAYLF

TpMuguga_03g00928 GGFFNPVSPTCNWGAKN-------ELPWIPGV---PLGQGYHWHLTDLVIPTMIILAYLF

TpMuguga_03g00913 GGFFNPVSPTCNWGAKN-------EPVWLPS----DLGSGYHWHLTDLVIPTMIILAYLF

TpMuguga_03g00616 GDFFDPVSPTCNWGAKN-------EPVWLPS----DLGTGYHWHLTDLVIPTMIILAYLF

TpMuguga_03g00908 GGFFDPVSPTCNWGATN-------KPVWLPGV---PLGQGYHWHLTDLVIPTMIILAYLF

TpMuguga_03g00916 GW-----SPKAAWTDNS-------KDFFPGS------PCNYMWHFFDLVIPTMIILAYLF

TpMuguga_03g00915 GW-----SPKAAWTDNS-------KDFFPGS------PCNYMWHFFDLVIPTMIILAYLF

TpMuguga_03g00927 GW-----SPKAAWTDNS-------KDFFPGS------PCNYMWHFFDLVIPTMIILAYLF

TpMuguga_03g00926 GW-----SPKAAWTDNS-------KDFFPGS------PCNYMWHFFDLVIPTMIILAYLF

TpMuguga_03g00906 GGFSSAWAPTTKWSGET-------TLNWGQTWNYDPNNFSFLWHITDLVIPTMIILAYLF

TpMuguga_03g00925 GGFSSAWAPTTKWSGET-------TLNWGQTWNYDPNNFSFLWHITDLVIPTMIILAYLF

TpMuguga_03g00917 GGFFNPVSPTCNWGAKN-------EAPWLPS----DLGSGYHWHLTDLVIPIMIILAYLF

TpMuguga_03g00918 GW-----SPKAEWKDNS-------GDPWAPG-----SPCNYLWHFFDLVIPTMIILAYLF

TpMuguga_03g00021 -----TESPKAEWAKPS--------VVWEPN------TDNRFWHTFILIPPFQICLTVIF

TpMuguga_03g02320 ----------GEWSVKYYEYPS--FQRWSQY------GAGNFYHFLVTLAPLQICLAIIF

TpMuguga_03g00563 ----------SEWSVKYYKYNSGGFQKWSQY------GAGNFYHFLVTLAPLQICLAIIF

TpMuguga_04g02490 ------------------------------------------------------------

TpMuguga_04g00590 -----YWHPMSKWT-----------------------GNGWFWHIFDIFIPLKIFLAVIF

TpMuguga_01g00590 -----KYSPKNKWE-----------------------DEKAYWHGTLIFIPVMGLCGILF

TpMuguga_02g00841 -----VYSPKNKWE-----------------------GGKEYWHGTLLFIPVMCICGFLF

TpMuguga_04g00058 -----NYSPKNQWS-----------------------GEKAYWNLTLIFIPIMIICGYLF

TpMuguga_01g00670 -----DYSPKNKWD-----------------------GDKVYWHGTLIFIPVMCICGFLF

TpMuguga_02g00477 -----GYGPDGKWE------------------------CRRHWHAFVIFIVSMIVCSILF

TpMuguga_03g00711 -----NYGPNKPWK-----------------------GGNSFWHGFIIFVIIEIAVGIMI

TpMuguga_03g00580 -----NLGPNKNWR-----------------------HSHSFWNFSALLIVPFLLMPILF

TpMuguga_02g00232 -----NIGPNCDWS------------------------VKKYWHLIWLFAIPYFICIVLF

**Supplemental Figure 3a, continued**

TpMuguga_03g00614 IYSLHYRDSSVARSIINQPKMSTCLTILFYMCHEISLAVGFPGI----------------

TpMuguga_03g00910 IYSLHYRDSSVARSIINQPKMSTCLTILFYMCHEISLAVGFPGI----------------

TpMuguga_03g00615 IYSLHYRDSSVARSIINQPKMSTCLTILFYMCHEISLAVGFPGI----------------

TpMuguga_03g00920 IYSLHYRDSSVARSIINQPKMSTCLTILFYMCHEISLAVGFPGI----------------

TpMuguga_03g00617 IYSLHYRDSSVARSIINQPKMSTCLTILFYMCHEISLAVGFPGI----------------

TpMuguga_03g00905 IYSLHYRDSSVARSIINQPKMSTCLTILFYMCHEISLAVGFPGI----------------

TpMuguga_03g00907 IYSLHYRDSSVARSIINQPKMSTCLTILFYMCHEISLAVGFPGI----------------

TpMuguga_03g00922 IYSLHYRDSSVARSIINQPKMSTCLTILFYMCHEISLAVGFPGI----------------

new_Tpr_chr_3_gap IYSLHYRDSSVARSIINQPKMSTCLTILFYMCHEISLAVGFPGI----------------

TpMuguga_03g00921 IYSLHYRDSSVARSIINQPKMSTCLTILFYMCHEISLAVGFPGI----------------

TpMuguga_03g00909 IYSLHYRDSSVARSIINQPKMSTCLTILFYMCHEISLAVGFPGI----------------

TpMuguga_03g00923 IYSLHYRDSSVARSIINQPKMSTCLTILFYMCHEISLAVGFPGI----------------

TpMuguga_03g00911 IYSLHYRDSSVARSIINQPKMSTCLTILFYMCHEISLAVGFPGI----------------

TpMuguga_03g00912 IYSLHYRDSSVARSIINQPKMSTCLTILFYMCHEISLAVGFPGI----------------

TpMuguga_03g00919 IYSLHYRDSSVARSIINQPKMSTCLTILFYMCHEISLAVGFPGI----------------

TpMuguga_03g00924 IYSLHYRDSSVARSIINQPKMSTCLTILFYMCHEISLAVGFPGI----------------

TpMuguga_03g00914 IYSLHYRDSSVARSIINQPKMSTCLTILFYMCHEISLAVGFPGI----------------

TpMuguga_03g00928 IYSLHYRDSSVARSIINQPKMSTCLTILFYMCHEISLAVGFPGI----------------

TpMuguga_03g00913 IYSLHYRDSSVARSIINQPKMSTCLTILFYMCHEISLAVGFPGI----------------

TpMuguga_03g00616 IYSLHYRDSSVARSIINQPKMSTCLTILFYMCHEISLAVGFPGI----------------

TpMuguga_03g00908 IYSLHYRDSSVARSIINQPKMSTCLTILFYMCHEISLAVGFPGI----------------

TpMuguga_03g00916 IYSLHYRDSSVARSIINQPKMSTCLTILFYMCHEISLAVGFPGI----------------

TpMuguga_03g00915 IYSLHYRDSSVARSIINQPKMSTCLTILFYMCHEISLAVGFPGI----------------

TpMuguga_03g00927 IYSLHYRDSSVARSIINQPKMSTCLTILFYMCHEISLAVGFPGI----------------

TpMuguga_03g00926 IYSLHYRDSSVARSIINQPKMSTCLTILFYMCHEISLAVGFPGI----------------

TpMuguga_03g00906 IYSLHYRDSSVARSIINQPKMSTCLTILFYMCHEISLAVGFPGI----------------

TpMuguga_03g00925 IYSLHYRDSSVARSIINQPKMSTCLTILFYMCHEISLAVGFPGI----------------

TpMuguga_03g00917 IYSLHYRDSSVARSIINQPKMSTCLTILFYMCHEISLAVGFPGL----------------

TpMuguga_03g00918 IYSLHYRDSSVARSIINQPKMSTCLTILFYMCHEILDNFT--------------------

TpMuguga_03g00021 IYSLHYRESSLSRSIINQPKTSTCLAILFYMCHEIQLAVGFPGM----------------

TpMuguga_03g02320 VYSLHHRESNVARSIINQPKMSTFLTILFYMCHECMLALGFSGF----------------

TpMuguga_03g00563 VYSLHHRESNVARSIINQPKMSTFLTILFYMCHECMLALGFSGF----------------

TpMuguga_04g02490 ------------------------------------------------------------

TpMuguga_04g00590 IYSLHHRDSRIARSIINKPKMTTTLTTIFYMCHSILLAVGFTGI----------------

TpMuguga_01g00590 IKALHYPYSGASLAIVNKPGMVGFLTILFYVSHMILLAVGYAGV----------------

TpMuguga_02g00841 IRALHYPYSGASLAIVNKPGMVGFLTILFYVSHIVLLAVGYAGV----------------

TpMuguga_04g00058 IRALHYPYSAASLAIMNKPGVVGFLAILFYVSHMVLLSIGYPGV----------------

TpMuguga_01g00670 IRALHYPFSGTSLAIVNKPGMVGFLTILFYVSHMILLAVGFPGV----------------

TpMuguga_02g00477 TTSLHYPHSDVGRSIVNKPMMTGFLTILFYISHEIMLSVGFPGI----------------

TpMuguga_03g00711 IFSLHHKHTALARSIIGKPVMASFLTMTYFTCHIIAIGVGFPGV----------------

TpMuguga_03g00580 IVPRHYPDTKLAELMTKNKAFLGFFTILFVVLHAVLVTVGYSAASMQT------------

TpMuguga_02g00232 LVPIHYPESHFGQLMTSNTAVLATVCFTFSISHAILKTVGFTGAGVQSTTIPNPTIVNNT

**Supplemental Figure 3a, continued**

TpMuguga_03g00614 -------------------------FGGNGGGSIL-ALTAQLIGAFLMCLLAPYSEGYII

TpMuguga_03g00910 -------------------------FGGNGGGSIL-ALTAQLIGAFLMCLLAPYSEGYII

TpMuguga_03g00615 -------------------------FGGNGGGSIL-ALTAQLIGAFLMCLLAPYSEGYII

TpMuguga_03g00920 -------------------------FGGNGGGSIL-ALTAQLIGAFLMCLLAPYSEGYII

TpMuguga_03g00617 -------------------------FGGNGGGSIL-ALTAQLMGAFLMCLLAPYSEGYII

TpMuguga_03g00905 -------------------------FGGNGGGSIL-ALTAQLMGAFLMCLLAPYSEGYII

TpMuguga_03g00907 -------------------------FGGNGGGSIL-ALTAQLMGAFLMCLLAPYSEGYII

TpMuguga_03g00922 -------------------------FGGNGGGSIL-ALTAQLMGAFLMCLLAPYSEGYII

new_Tpr_chr_3_gap -------------------------FGGNGGGSIL-ALTAQLMGAFLMCLLAPYSEGYII

TpMuguga_03g00921 -------------------------FGGNGGGSIL-ALTAQLMGAFLMCLLAPYSEGYII

TpMuguga_03g00909 -------------------------FGGNGGGSIL-ALTAQLMGAFLMCLLAPYSEGYII

TpMuguga_03g00923 -------------------------FGGNGGGSIL-ALTAQLMGAFLMCLLAPYSEGYII

TpMuguga_03g00911 -------------------------FGGNGGGSIL-ALTAQLMGAFLMCLLAPYSEGYII

TpMuguga_03g00912 -------------------------FGGNGGGSIL-ALTAQLIGAFLMCLLAPYSEGYII

TpMuguga_03g00919 -------------------------FGGNGGGSIL-ALTAQLIGAFLMCLLAPYSEGYII

TpMuguga_03g00924 -------------------------FGGNGGGSIL-ALTAQLIGAFLMCLLAPYSEGYII

TpMuguga_03g00914 -------------------------FGGNGGGSIL-ALTAQLMGAFLMCLLAPYSEGYII

TpMuguga_03g00928 -------------------------FGGNGGGSIL-ALTAQLMGAFLMCLLAPYSEGYII

TpMuguga_03g00913 -------------------------FGGNGGGSIL-ALTAQLIGAFLMCLLAPYSEGYII

TpMuguga_03g00616 -------------------------FGGNGGGSIL-ALTAQLMGAFLMCLLAPYSEGYII

TpMuguga_03g00908 -------------------------FGGNGGGSIL-ALTAQLIGAFLMCLLAPYSEGYII

TpMuguga_03g00916 -------------------------FGGNGGGSIL-ALTAQLMGAFLMCLLAPYSEGYII

TpMuguga_03g00915 -------------------------FGGNGGGSIL-ALTAQLIGAFLMCLLAPYSEGYII

TpMuguga_03g00927 -------------------------FGGNGGGSIL-ALTAQLIGAFLMCLLAPYSEGYII

TpMuguga_03g00926 -------------------------FGGNGGGSIL-ALTAQLIGAFLMCLLAPYSEGYII

TpMuguga_03g00906 -------------------------FGGNGGGSIL-ALTAQLIGAFLMCLLAPYSEGYII

TpMuguga_03g00925 -------------------------FGGNGGGSIL-ALTAQLIGAFLMCLLAPYSEGYII

TpMuguga_03g00917 -------------------------LGNSGFSSIL-ALMAQLMGALFGF-----------

TpMuguga_03g00918 ------------------------------------------------------------

TpMuguga_03g00021 -------------------------VGNKGGDHVM--LPTQYAGALLMIFLALYSEGYIT

TpMuguga_03g02320 -------------------------IGNKGGDLI---LIPQYIGALIMIFGAMYSEGYII

TpMuguga_03g00563 -------------------------IGNKGGDLI---LIPQYIGALIMIFGAMYSEGYII

TpMuguga_04g02490 -------------------------------------LPITLISTILMILLTFYSQGYIT

TpMuguga_04g00590 -------------------------IGNGGANYFL--LPTQFSGALLSVFLEFYSIGYVD

TpMuguga_01g00590 -------------------------GPNWNSTGS---TVNGFLTNLSMILLIFLAEGYIN

TpMuguga_02g00841 -------------------------GPNWNSTGS---TVNGFLTNLSMILLIFLAEGYIN

TpMuguga_04g00058 -------------------------EKNWNSTGS---TVTGFLATFAMIIFVFLSEGYIN

TpMuguga_01g00670 -------------------------EKNSGGAKDYLPTINGFLTTGAMIVFLFLSEGYIN

TpMuguga_02g00477 -------------------------SENWNSNAT---TGTGLLCGTGLMIFVLFGEGYII

TpMuguga_03g00711 -------------------------DANSNGTIG---TVNLFLSLLFMNLLELLGEGYVV

TpMuguga_03g00580 -------------------------NDQYNSSMT---AFNTFLSYFFTVFIVLLSQGYLK

TpMuguga_02g00232 DIQFNPRGSDAPQLTVGKGKPNGRKDRSRNGQVS---SFNILLSYFFLVFFAFLGDGYIK

**Supplemental Figure 3a, continued**

TpMuguga_03g00614 EYKRH--DPSNWPTAGMTRWNALRYWTKMASKNCNKNLAALFTKDLRRDLL

TpMuguga_03g00910 EYKRH--DPSNWPTAGMTRWNALRYWTKQASKNCNKNLAALFTKDLRRDLL

TpMuguga_03g00615 EYKRH--DPSNWPTAGMTRWNALRYWTKMASKNCNKNLAALFTKDLRRDLL

TpMuguga_03g00920 EYKRH--DPSNWPTAGMTRWNALRYWTKMASKNCNKNLAALFTKDLRRDLL

TpMuguga_03g00617 EYKRH--DPSNWPTAGMTRWNALRYWTKMASKNCNKNLAALFTKDLRRDLL

TpMuguga_03g00905 EYKRH--DPSNWPTAGMTRWNALRYWTKMASKNCNKNLAALFTKDLRRDLL

TpMuguga_03g00907 EYKRH--DPSNWPTAGMTRWNALRYWTKMASKNCNKNLAALFTKDLRRDLL

TpMuguga_03g00922 EYKRH--DPSNWPTAGMTRWN-LRYWTKMASKNCNKNLAALFTKDLRRDLL

new_Tpr_chr_3_gap EYKRH--DPSNWPTAGMTRWNALRYWTKMASKNCNKNLAALFTKDLRRDLL

TpMuguga_03g00921 EYKRH--DPSNWPTAGMTRWNALRYWTKMASKNCNKNLAALFTKDLRRDLL

TpMuguga_03g00909 EYKRH--DPSNWPTAGMTRWNALRYWTKQASKNCNKNLAALFTKDLRRDLL

TpMuguga_03g00923 EYKRH--DPSNWPTAGMTRWNALRYWTKQASKNCNKNLAALFTKDLRRDLL

TpMuguga_03g00911 EYKRH--DPSNWPTAGMTRWNALRYWTKQASKNCNKNLAALFTKDLRRDLL

TpMuguga_03g00912 EYKRH--DPSNWPTAGMTRWNALRYWTKMASKNCNKNLAALFTKDLRRDLL

TpMuguga_03g00919 EYKRH--DPSNWPTAGMTRWNSLRYWTKMASKNCNKNLAALFTKDLRRDLL

TpMuguga_03g00924 EYKRH--DPSNWPTAGMTRWNALRYWTKQASKNCNKNLAALFTKDLRRDLL

TpMuguga_03g00914 EYKRH--DPSNWPTAGMTRWNALRYWTKQASKNCNKNLAALFTKDLRRDLL

TpMuguga_03g00928 EYKRH--DPSNWPTAGMTRWNALRYWTKQASKNCNKNLAALFTKDLRRDLL

TpMuguga_03g00913 EYKRH--DPSNWPTAGMTRWNALRYWTKMASKNCNKNLAALFTKDLRRDLL

TpMuguga_03g00616 EYKRH--DPSNWPTAGMTRWNALRYWTKQASKNCNKNLAALFTKDLRRDLL

TpMuguga_03g00908 EYKRH--DPSNWPTAGMTRWNALRYWTKQASKNCNKNLAALFTKDLRRDLL

TpMuguga_03g00916 EYKRH--DPSNWPTAGMTRWNALRYWTKMASKNCNKNLAALFTKDLRRDLL

TpMuguga_03g00915 EYKRH--DPSNWPTAGMTRWNALRYWTKMASKNCNKNLAALFTKDLRRDLL

TpMuguga_03g00927 EYKRH--DPSNWPTAGMTRWNALRYWTKMASKNCNKNLAALFTKDLRRDLL

TpMuguga_03g00926 EYKRH--DPSNWPTAGMTRWNALRYWTKQASKNCNKNLAALFTKDLRRDLL

TpMuguga_03g00906 EYKRH--DPSNWPTAGMTRWNALRYWTKQASKNCNKNLAALFTKDLRRDLL

TpMuguga_03g00925 EYKRH--DPSNWPTAGMTRWNALRYWTKQASKNCNKNLAALFTKDLRRDLL

TpMuguga_03g00917 ---------------------------------------------------

TpMuguga_03g00918 ---------------------------------------------------

TpMuguga_03g00021 EYKRH--DPSRWPTTGMTKWNAFCYWTKRASKICNHNIASLFTRDLRKDLL

TpMuguga_03g02320 EYKKH--DPKNWPTAGMTGWNAFKYWAKMGSKNCNHNLKHLFTKDLRRDLL

TpMuguga_03g00563 EYNKL----------------------------------------------

TpMuguga_04g02490 EYKRH--SHNSWPTDDMTTWNALCYWLKKSHKITKHNLKSLFTTNLRSDLL

TpMuguga_04g00590 VYKRH--DPLRWPTDGMSSLKAFGYWTKMACNSSKNHLRDLFTKDLRRDLL

TpMuguga_01g00590 EFKKH--YRAYWPTTGLSNKRAFGFWFDKALQNGFKNIMMIFTRDLRRDLL

TpMuguga_02g00841 EFKKH--DRAYWPTTGLSNRRAFGFWFDKALQNGFKNLMAIFTRDLRRDLL

TpMuguga_04g00058 EFKKH--DRAYWPTTGLSNRRAFGFWFDKALQNGFKNFLLIFSRDLRRDLL

TpMuguga_01g00670 EYKKH--DFANWPTEGLSNKRALGFWFDKALQNGWKNFKLIFTRDLRRDLM

TpMuguga_02g00477 EYKRH--NKLKWPTDGMATRTAFNYWMSRSFANAVDNVKCIFTSDVRRDIL

TpMuguga_03g00711 EYKKY—TETTWPTGGMTDCEALRFWLRRAAANAWISLKSSVTTDVRRKLL

TpMuguga_03g00580 TYKDYLNNIDTWATNDMKGPRAFFYWLGKSFEHGWEAFTETFSSDIKSDIL

TpMuguga_02g00232 TIKLFERNRDFWPTYDMGFFASFAFWTKKSFKRGLKSFGEVFTLNVRDQLM

b.


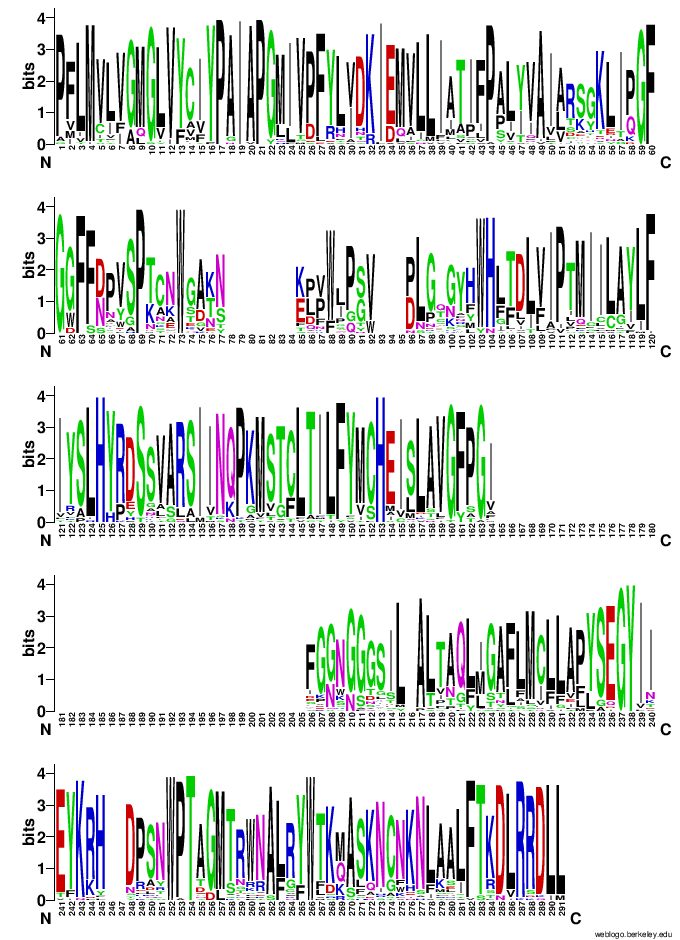


Supplemental Figure 3: Amino acid sequence alignment of the conserved domain of Tpr family proteins in *T. parva*.

(a). The alignment was used to build an HMM profile to search other *Theileria* species for detection of orthologs. The conserved domain, which codes for five transmembrane helices, is present in all proteins annotated as “Tpr family protein”. (b). A webLogo was generated using the sequence alignment in Supplemental Figure 3a. The values along the x-axis in each row correspond to the position in the sequence and the bits on the y-axis measures the height of each letter, which is relative to the frequency of the amino acid in the sequence.

#

#

#

#

#

#

#

#

#

#

# Supplemental Figure 4: Phylogenetic trees of full-length nucleotide sequences for genes used to reconstruct the species phylogeny, shown as inset phylogeny in Figure 3.

Ten othologous, conserved, singly-copy genes were selected from for phylogenetic analysis. Each gene was submitted to MAFFT, which indicated, in all cases, the L-INS-I alignment algorithm as best fit for each dataset. Alignments were then submitted to modeltest-nj to select the best-fitting model of evolution. Alignments and the selected model were submitted to RAxML for a “quick-and-dirty” analysis which called on autoMRE to stop bootstrapping replicates (BS) once log likelihood scores converged or maximum replications were reached. The ten genes (the *T. parva* ortholog is listed) are the following:

(a) TpMuguga_01g00845, encoding 26S proteasome subunit 4: 1,721 nucleotides in the reference allele of *T. parva* Muguga, including five introns, producing an alignment of 2,300 positions, with GTR as the chosen best fitting model, and 1,000 BS replicates;

(b) TpMuguga_01g00924, encoding 60S ribosomal protein L31: 454 nucleotides in *T. parva* Muguga (2 introns), producing an alignment of 723 positions, with HKY85 as the chosen best fitting model, and 900 BS replicates;

(c) TpMuguga_01g00633, encoding Adenylate kinase 3: 1,156 nucleotides in *T. parva* Muguga (4 introns), and producing an alignment of 2,135 positions, with HKY85 as the chosen best fitting model, and 1,000 BS replicates;

(d) TpMuguga_03g00773, encoding Adenylosuccinate lyase: 1,780 nucleotides in *T. parva* Muguga (7 introns), producing an alignment of 2,357 positions, with HKY85 as the chosen best fitting model, and 1,000 BS replicates;

(e) TpMuguga_04g00620, encoding Isocitrate dehydrogenase: 1,649 nucleotides in *T. parva* Muguga (5 introns) and producing an alignment of 1,888 positions, with GTRGAMMA as the chosen best fitting model, and 1,000 BS replicates;

(f) TpMuguga_01g00500, encoding Myosin A: 2,671 nucleotides in *T. parva* Muguga (2 introns), producing an alignment of 3,584 positions, with GTR as the chosen best fitting model, and 950 BS replicates;

(g) TpMuguga_01g00188, encoding Prohibitin: 1,048 nucleotides in *T. parva* Muguga (1 intron), producing an alignment of 1,221 positions, with GTR as the chosen best fitting model, and 1,000 BS replicates;

(h) TpMuguga_04g00678, encoding Calcium-dependent protein kinase 4: 1,577 nucleotides in *T. parva* Muguga (1 intron), producing an alignment of 2,000 positions, with GTR as the chosen best fitting model, and 1,000 BS replicates;

(i) TpMuguga_04g00748, encoding Ribosomal protein S2: 955 nucleotides in *T. parva* Muguga (1 intron), producing an alignment of 1,198 positions, with GTR as the chosen best fitting model, and 1,000 BS replicates;

(j) TpMuguga_04g02265, Signal recognition particle subunit SRP68: 1,994 nucleotides in *T. parva* Muguga (9 introns), producing an alignment of 2,281 positions, with HKY85 as the chosen best fitting model, and 300 BS replicates.

The 10 alignments were concatenated and a partitioned alignment model was submitted to RAxML for three independent and rigorous analyses (inset phylogeny in Figure 3).


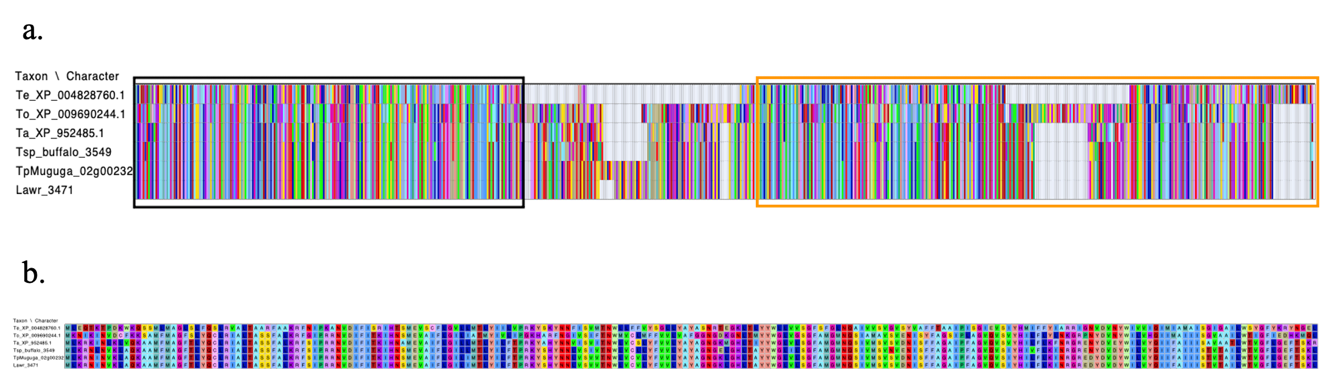


Supplemental Figure 5: Example of a conserved domain across all *Theileria* species orthologs.

There were several instances where *Tpr* orthologs formed a monophyletic clade, and the sequences followed the same pattern of relationship as the species from which they were obtained. We show one example of this, aligning the sequences within the clade α, containing TpMuguga_02g00232 (a), which shows the conserved N-terminal segment within the black box and the conserved C-terminal segment within the orange box. The aligned sequences of only the conserved N-terminal region of the protein is shown, with the amino acid sequence visible (b).

# Supplemental Tables

# Supplemental Table 1. Coordinates of new genes identified as a result of closing gaps in *T. parva* reference genome.

| Locus_tag | Contig | Start | End | Strand | Notes |
| --- | --- | --- | --- | --- | --- |
| Chr.3 | | | | | |
| TpMuguga_03g00614 | unitig_5 | 568,144 | 570,110 | - | Completion of partial |
| TpMuguga_03g02700 | unitig_5 | 570,356 | 572,316 | - | New gene |
| TpMuguga_03g00922 | unitig_5 | 572,985 | 574,937 | - | Completion of partial |
| Chr.4 | | | | | |
| TpMuguga_04g02720 | unitig_2 | 40,644 | 42,236 | + | New gene |
| TpMuguga_04g02725 | unitig_2 | 42,525 | 44,223 | + | New gene |
| TpMuguga_04g02730 | unitig_2 | 44,433 | 45,968 | + | New gene |
| TpMuguga_04g02735 | unitig_2 | 46,257 | 47,585 | + | New gene |
